# Normal tissue homeostasis and impairment of selective inflammatory responses in dendritic cells deficient for ATF6α

**DOI:** 10.3389/fcell.2023.1089728

**Published:** 2023-03-21

**Authors:** Francisca Gutiérrez-Ballesteros, Jonathan Morales-Reyes, Dominique Fernández, Antonia Geisse, Amada Arcaya, Felipe Flores-Santibañez, María Rosa Bono, Fabiola Osorio

**Affiliations:** ^1^ Laboratory of Immunology and Cellular Stress, Immunology Program, Institute of Biomedical Sciences, Faculty of Medicine, University of Chile, Santiago, Chile; ^2^ Immunology Laboratory, Biology Department, Faculty of Sciences, University of Chile, Santiago, Chile

**Keywords:** dendritic cells, unfolded protein response, ATF6, tissues, immunity, proinflammatory cytokines, IL-12, IL-6

## Abstract

The initiation of adaptive immunity relies on the performance of dendritic cells (DCs), which are specialized leukocytes with professional antigen presenting capabilities. As such, the molecular mechanisms safeguarding DC homeostasis are matter of intense research. Sensors of the unfolded protein response (UPR) of the endoplasmic reticulum, a three-pronged signaling pathway that maintains the fidelity of the cellular proteome, have emerged as regulators of DC biology. The archetypical example is the IRE1/XBP1s axis, which supports DC development and survival of the conventional type 1 DC (cDC1) subtype. However, the role of additional UPR sensors in DC biology, such as the ATF6α branch, has not been clearly elucidated. Even though *Xbp1* is transcriptionally induced by ATF6α under ER stress, it is unclear if cDCs also co-opt the ATF6α branch in tissues. Here, we examine the role of ATF6α in cDC homeostasis *in vivo* and upon innate stimulation *in vitro*. In steady state, animals lacking ATF6α in CD11c^+^ cells (*Itgax* Cre x *Atf6* ^fl/fl^ mice) display normal cDC frequencies in spleen, intestine, liver, and lung. Also, ATF6α deficient cDCs express normal levels of *Xbp1* mRNA and additional UPR components. However, a reduction of lung monocytes is observed in *Itgax* Cre x *Atf6* ^fl/fl^ conditional deficient animals suggesting that ATF6α may play a role in the biology of monocyte subsets. Notably, in settings of DC activation, ATF6α contributes to the production of IL-12 and IL-6 to inflammatory stimuli. Thus, although ATF6α may be dispensable for tissue cDC homeostasis in steady state, the transcription factor plays a role in the acquisition of selective immunogenic features by activated DCs.

## Introduction

Dendritic cells (DCs) are chief sentinels of the immune system responsible to couple innate and adaptive immunity (Cabeza-Cabrerizo et al., 2021). DCs are a heterogeneous family of leukocytes that include plasmacytoid DCs (pDCs, known to promote antiviral immunity), and conventional DCs (cDC), which are divided into type 1 cDCs (cDC1) and type 2 cDCs (cDC2) (Murphy et al., 2016). Due to their heightened capacity to activate CD8^+^ T cells against tumors and virally-infected cells, cDC1s have become central targets in immunotherapy whereas cDC2s are prone to activate CD4^+^ T cells against extracellular bacteria, fungi, and parasites (Murphy et al., 2016).

The capacity of DCs to orchestrate antigen specific immune responses has fostered scientific efforts to better understand molecular mechanisms safeguarding DC function. An emerging intracellular pathway regulating DC biology is the unfolded protein response (UPR), a response that maintains the fidelity of the cellular proteome in conditions eliciting endoplasmic reticulum (ER) stress, such as in infection, chronic inflammation and metabolic dysregulation (Grootjans et al., 2016; Smith et al., 2020). The UPR is initiated by three ER resident sensors: PERK (protein kinase R-like ER kinase), IRE1 (inositol-requiring enzyme 1, Alpha) and ATF6 (Activating transcription factor 6). PERK activation promotes attenuation of global protein translation, selective activation of amino acid metabolism/oxidative stress genes and the coordination of cell death *via* the pro-apoptotic transcription factor CHOP (Grootjans et al., 2016; Hetz et al., 2020). IRE1 is an enzyme bearing a serine-threonine kinase and endoribonuclease (RNase) domain, which mediates unconventional splicing of *Xbp1*u mRNA (X box binding protein 1, unspliced), prompting the translation of XBP1s (XBP1 spliced), a potent transcription factor and key activator of ER biogenesis, lipid biosynthesis and chaperone genes (Grootjans et al., 2016; Read and Schröder, 2021). In addition, in poorly defined conditions of ER stress, IRE1 RNase can degrade diverse mRNAs/microRNAs through a mechanism known as “regulated IRE1-dependent decay” (RIDD) (Hetz et al., 2020).

ATF6 is a member of the bZIP family with two homologous proteins, ATF6α (encoded by the *Atf6* gene) and ATF6β in mammals (Haze et al., 1999; Yoshida et al., 2001; Adachi et al., 2008; Almanza et al., 2019). ATF6α is a potent transcription factor known to control expression of genes coding for chaperones, lipid biosynthesis and ERAD (ER Associated Degradation) members in contexts of ER stress (Thuerauf et al., 2002; Yamamoto et al., 2004; Sharma et al., 2019). In contrast, the role of ATF6β is less understood, and it is proposed to possess weaker transcriptional activity than ATF6α (Yoshida et al., 1998; Yoshida et al., 2000; Thuerauf et al., 2004; Thuerauf et al., 2007). ATF6β has also shown to counteract ATF6α transcriptional activity (Thuerauf et al., 2004) and to date, ATF6α is the predominant isoform controlling cellular responses during ER stress settings (Glembotski et al., 2019).

Interestingly, the UPR branches can be also co-regulated to safeguard protein homeostasis (Shoulders et al., 2013). For instance, ATF6α controls expression of *Xbp1* (Yoshida et al., 2000; Yoshida et al., 2001), and ATF6α and XBP1s can also form heterodimers that regulate expression of selected proteostatic genes (Yamamoto et al., 2007; Shoulders et al., 2013; Vidal et al., 2021). Notably, despite this knowledge, the interplay between ATF6α and XBP1s has not been extended *in vivo* to tissue resident cells.

Regarding DC subtypes, pDCs and cDCs are highly sensitive to perturbations in UPR components and require IRE1/XBP1s signaling for development (Iwakoshi et al., 2007; Flores-Santibáñez et al., 2019). In differentiated stages, cDC1s display constitutive IRE1 RNase activity (Osorio et al., 2014) and selectively depend on IRE1/XBP1s signaling for survival in tissues such as the lung (Tavernier et al., 2017). PERK also controls certain DC/cDC1 functions, which is evidenced by high rate of eIF2α phosphorylation in steady state cDC1s (Mendes et al., 2020). Furthermore, in contexts of DC activation, the IRE1/XBP1s and PERK branches are critical to fine tune immunogenic features of activated DCs (Mogilenko et al., 2019). As such, DCs selectively activate UPR components but to date, there is no evidence addressing the role of ATF6α in DC biology. This is a relevant question considering that DC subtypes are increasingly studied in their capacity to fine tune UPR components to regulate immunity. Here, we studied cDC homeostasis in tissues from animals bearing selective deletion of ATF6α in DCs. Our data shows that mice lacking ATF6α in DCs display normal cDC composition in lymphoid and non-lymphoid organs. We also observe that ATF6α deficient cDCs expressed normal levels of *Xbp1s* and additional UPR components. However, ATF6α deficiency in CD11c^+^ cells resulted in reduced frequencies of lung monocytes, suggesting that the transcription factor may influence the biology of monocyte subtypes in tissues. Finally, in contexts of DC activation with inflammatory stimuli, our data reveal a contribution of ATF6α in the production of IL-12 and IL-6 by bone marrow-derived DCs. Altogether, our data indicate that the UPR sensor ATF6α does not control influence tissue DC homeostasis in steady state, but it selectively tunes the production of specific proinflammatory cytokines in contexts of activation.

## Results

### ATF6α deficiency does not alter cDC composition in the spleen

**FIGURE 1:**
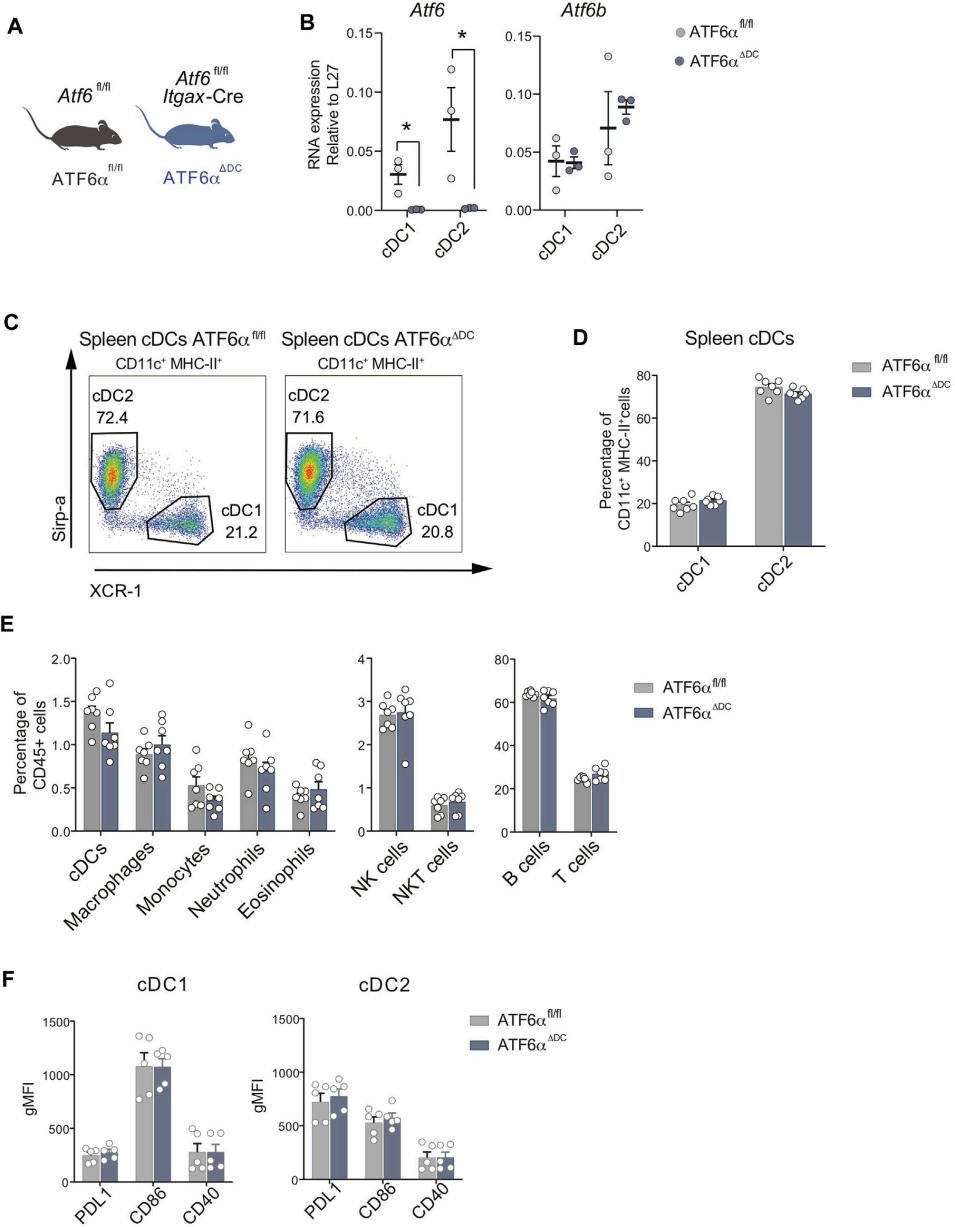
ATF6α deficiency does not alter cDC composition in spleen. **(A)** Schematic representation of ATF6α^ΔDC^ mouse model. **(B)** Quantification of L27-relative expression of *Atf6* and *Atf6b* mRNA in cDC1s and cDC2s sorted from spleen of ATF6α^fl/fl^ (*n* = 3 mice) and ATF6α^ΔDC^ mice (*n* = 3 mice). Quantification was performed by qPCR. Each symbol represents one mouse obtained from two independent experiments. **(C)** Representative flow cytometry plots showing distribution of cDC1s and cDC2s in spleen of ATF6α^fl/fl^ and ATF6α^ΔDC^ mice. Cells were pre-gated as single live CD45^+^, CD64^−^, F4/80^−^, B220^−^, CD3^−^, LY6G^−^, NK1.1^−^, Ly6C^−/int^, CD11b^+^, CD11c^+^, MHC-II^+^. **(D)** Percentage of cDC1s and cDC2s relative to CD11c^+^ MHC-II^+^ cells in spleen of ATF6α^fl/fl^ (*n* = 7 mice) and ATF6α^ΔDC^ mice (*n* = 7 mice). Bar graph depicts mean percentage of cells (±s.e.m.). Each symbol represents one sample obtained from 3 independent experiments. **(E)** Percentage of cDCs, macrophages, monocytes, neutrophils, eosinophils, NK, NKT, B and T cells relative to the total percentage of CD45^+^ cells in spleen of ATF6α^fl/fl^ (*n* = 7 mice) and ATF6α^ΔDC^ mice (*n* = 7 mice). Bar graph depicts mean percentage of cells (±s.e.m.). Each symbol represents one sample obtained from 3 independent experiments. **(F)** Quantification of costimulatory molecules in cDC1s and cDC2s from spleen of ATF6^fl/fl^ (*n* = 5 mice) and ATF6α^ΔDC^ mice (*n* = 5 mice). The identification of co-stimulatory molecules was carried out by labeling with antibodies. Each symbol represents the sample obtained from a mouse in three independent experiments. For statistical analyses in **(B–F)** a non-parametric Mann-Whitney test was used, *p < 0.05.

To obtain insights on the role of ATF6α in DC homeostasis, we generated conditional *knock-out* animals lacking ATF6α in CD11c-expressing cells. To this end, we crossed the *Itgax*-Cre mice line with *Atf6* ^fl/fl^ mice (referred to as “ATF6α^ΔDC^ mice”) (Engin et al., 2013). These animals delete exons 8-9 of *Atf6* in CD11c-expressing cells (which fully targets cDC1s and cDC2s, while partially targeting pDCs and monocyte/macrophage subsets (Abram et al., 2014)). ATF6α^ΔDC^ mice are compared to control animals (*Atf6* ^fl/fl^ littermates with no expression of Cre, scheme depicted in Figure 1A). Spleen cDCs were isolated from ATF6α^ΔDC^ mice and control animals and expression of ATF6α was quantified by qPCR (Figure 1B). As expected, cDC1s and cDC2s from ATF6α^ΔDC^ mice do not express *Atf6* mRNA and maintain normal expression of *Atf6b*, validating the model of study. Next, we analyzed the composition of cDC1s and cDC2s and observed that ATF6α^ΔDC^ mice display unaltered frequencies of these subtypes (Figures 1C, D). Furthermore, cellular composition analysis of ATF6α^ΔDC^ mice in spleen revealed that these animals have normal composition of immune cell types (Figure 1E, gating analysis in Supplementary Figure S1).

To assess whether ATF6α deficient cDCs undergo normal differentiation/activation, we quantified expression of the costimulatory molecules PD-L1, CD86 and CD40, which are surface immunoregulatory molecules that allow cDCs to restrain or activate T cells, respectively (Kapsenberg, 2003; Hubo et al., 2013). Data in Figure 1F show that ATF6α deficiency does not alter surface expression of these proteins in cDCs. In conclusion, ATF6α does not regulate the differentiation/activation program of steady state cDCs in spleen.

### Normal DC composition in the small intestine lamina propria and liver of ATF6α^ΔDC^ mice

**FIGURE 2:**
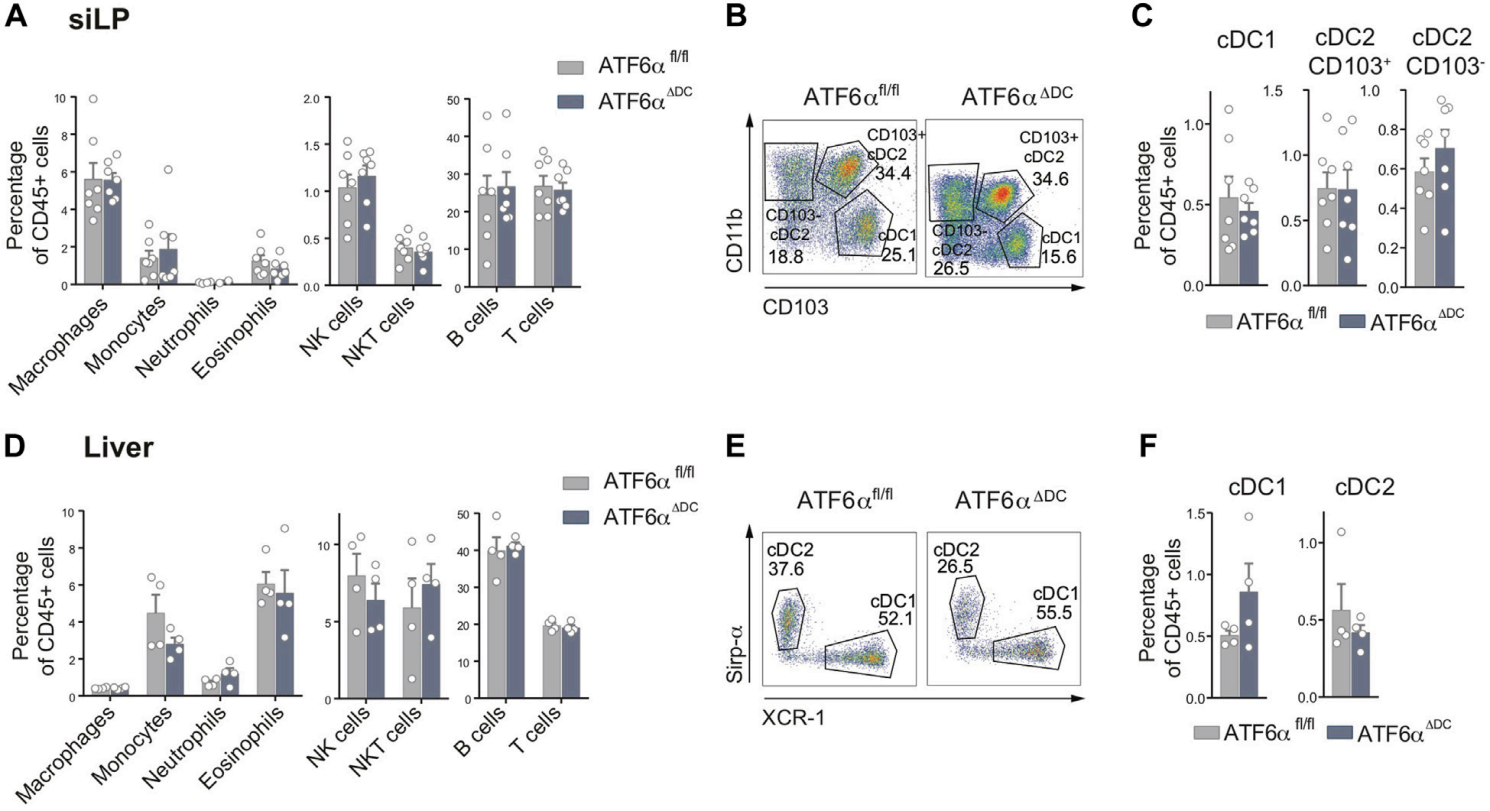
Normal cDC composition in small intestine lamina propria and liver of ATF6α-deficient mice. **(A)** Percentage of immune cells relative to CD45^+^ cells from SiLP of ATF6α^fl/fl^ (*n* = 7 mice) and ATF6α^ΔDC^ mice (*n* = 7 mice). Bar graph depicts mean percentage of cells (±s.e.m.). Each symbol represents one sample obtained from 3 independent experiments. **(B)** Representative flow cytometry plots showing distribution of cDC1s and cDC2 subsets in SiLP of ATF6α^fl/fl^ and ATF6α^ΔDC^ mice. Cells were pre-gated as single live CD45^+^, CD64^−^, F4/80^−^, B220^−^, CD3^−^, LY6G^−^, NK1.1^−^, Ly6C^−/int^, CD11b^+^, CD11c^+^, MHC-II^+^. **(C)** Percentage of cDC1s and cDC2s relative to CD45^+^ cells from SiLP of ATF6α^fl/fl^ (*n* = 7 mice) and ATF6α^ΔDC^ mice (*n* = 7 mice). Bar graph depicts mean percentage of cells (±s.e.m.). Each symbol represents one sample obtained from 3 independent experiments. **(D)** Percentage of immune cells relative to CD45^+^ cells from liver of ATF6α^fl/fl^ (*n* = 7 mice) and ATF6α^ΔDC^ mice (*n* = 7 mice). Bar graph depicts mean percentage of cells (±s.e.m.). Each symbol represents one sample obtained from 3 independent experiments. **(E)** Representative flow cytometry plots showing distribution of cDC1s and cDC2s in liver of ATF6^fl/fl^ and ATF6^ΔDC^ mice. Cells were pre-gated as single live CD45^+^, CD64^−^, F4/80^−^, B220^−^, CD3^−^, LY6G^−^, NK1.1^−^, Ly6C^−/int^, CD11b^+^, CD11c^+^, MHC-II^+^. **(F)** Percentage of cDC1s and cDC2s relative to CD45^+^ cells from liver of ATF6α^fl/fl^ (*n* = 7 mice) and ATF6α^ΔDC^ mice (*n* = 7 mice). Bar graph depicts mean percentage of cells (±s.e.m.). Each symbol represents one sample obtained from 3 independent experiments. For statistical analyses in **(A,C,D, and F)**, non-parametric Mann-Whitney test was used.

Considering that cDCs in lymphoid organs are not equivalent to counterparts exposed to inflammatory stimuli in non-lymphoid tissues, we analyzed cDCs from the small intestine lamina propria (SiLP) and liver of ATF6α^ΔDC^ mice (Figure 2, gating analysis Supplementary Figures S1A, B). We verified that archetypical immune cell types were present in normal frequencies in the SiLP of ATF6α^ΔDC^ mice (Figure 2A). In the SiLP, *bona-fide* cDCs are divided in cDC1s (defined as CD103^+^CD11b^−^) and two subsets of cDC2s (CD103^+^CD11b^+^ and CD103^−^CD11b^+^) (Sun et al., 2020). Analysis of ATF6α^ΔDC^ mice show normal frequencies of the three cDC subtypes at the SiLP (Figures 2B–C). Liver tissue analysis show similar results, normal immune cell composition (Figure 2D, gating analysis in Supplementary Figure S2B) and comparable cDC frequencies between ATF6α deficient and control counterparts (Figures 2E, F). Altogether, these data indicate that ATF6α loss does not impair cDC homeostasis in tissues.

### Deletion of ATF6α does not recapitulate XBP1 deficiency in lung cDCs

**FIGURE 3:**
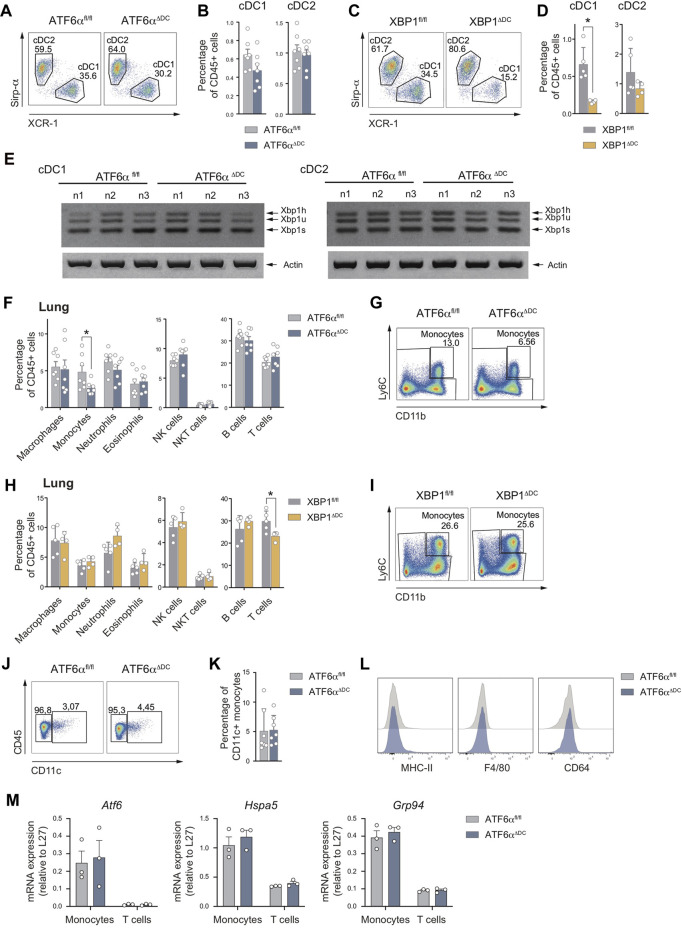
The loss of lung cDC1s observed upon XBP1 deletion is not recapitulated by deficiency of ATF6α **(A)** Representative flow cytometry plots showing distribution of cDC1s and cDC2s in lung of ATF6α^fl/fl^ and ATF6α^ΔDC^ mice. **(B)** Percentage of cDC1s and DC2s relative to CD45^+^ cells from lung of ATF6α^fl/fl^ (*n* = 7 mice) and ATF6α^ΔDC^ mice (*n* = 7 mice). Bar graph depicts mean percentage of cells (±s.e.m.). Each symbol represents one sample obtained from 3 independent experiments. **(C)** Representative flow cytometry plots showing distribution of cDC1s and cDC2s in lung of XBP1^fl/fl^ and XBP1^ΔDC^ mice. **(D)** Percentage of cDC1s and DC2s relative to CD45^+^ cells from lung of XBP1^fl/fl^ (*n* = 5 mice) and XBP1^ΔDC^ mice (*n* = 4 mice). Bar graph depicts mean percentage of cells (±s.e.m.). Each symbol represents one sample obtained from two independent experiment. **(E)** RT-PCR analysis of *Xbp1* splicing in cDC1s and cDC2s sorted from spleen of ATF6α^fl/fl^ (*n* = 3 mice) and ATF6α^ΔDC^ mice (*n* = 3 mice). Data were obtained from two independent experiments. *Xbp1h: Xbp1* hybrid, *Xbp1u: Xbp1* unspliced, *Xbp1s: Xbp1* spliced. Actin (bottom) serves as a loading control. **(F)** Percentage of immune cells relative to CD45^+^ cells from lung of ATF6α^fl/fl^ (*n* = 7 mice) and ATF6α^ΔDC^ mice (*n* = 7 mice). Bar graph depicts mean percentage of cells (±s.e.m.). Each symbol represents one sample obtained from 3 independent experiments. **(G)** Representative flow cytometry plots showing monocytes from lung of ATF6α^fl/fl^ and ATF6α^ΔDC^ mice. **(H)** Percentage of immune cells relative to CD45^+^ cells from lung of XBP1^fl/fl^ (*n* = 5 mice) and XBP1^ΔDC^ mice (*n* = 4 mice). Bar graph depicts mean percentage of cells (±s.e.m.). Each symbol represents one sample obtained from two independent experiment. **(I)** Representative flow cytometry plots showing lung monocytes from XBP1^fl/fl^ and XBP1^ΔDC^ mice. **(J)** Representative flow cytometry plots showing CD11c expression by lung monocytes from ATF6α^fl/fl^ and ATF6α^ΔDC^ mice. **(K)** Percentage of CD11c^+^ lung monocytes from ATF6α^fl/fl^ (*n* = 7 mice) and ATF6α^ΔDC^ mice (n = 7 mice). Bar graph depicts mean percentage of cells (±s.e.m.). Each symbol represents one sample obtained from 3 independent experiments. **(L)** Representative flow cytometry plots showing MHC-II, F4/80 and CD64 expression by lung monocytes from ATF6α^fl/fl^ and ATF6α^ΔDC^ mice. **(M)** Quantification of *Atf6*, *Hspa5* and *Grp94* expression in monocytes and T cells sorted from lung of ATF6α^fl/fl^ (*n* = 3 mice) and ATF6α^ΔDC^ mice (*n* = 3 mice) by qPCR. Each symbol represents one mouse. For statistical analyses in **(B,D,F,H,K, and M)**, non-parametric Mann-Whitney test was used.

The *Xbp1* gene is a transcriptional ATF6α target that contains an ER stress response element (ERSE) consensus sequence on its promoter region (Yoshida et al., 2000). Phenotypically, XBP1s deficiency in DCs leads to a marked reduction in cDC1 frequencies in the lung (Tavernier et al., 2017). The interplay between ATF6α and XBP1s led us to hypothesize that ATF6α^ΔDC^ mice may recapitulate the loss of lung cDC1s observed in XBP1 conditional deficient mice. To test this hypothesis, we generated conditional *knock-out* animals lacking XBP1s in CD11c-expressing cells by crossing the *Itgax*-Cre mice line with *Xbp1* ^fl/fl^ mice (Lee et al., 2008) (referred to as “XBP1^ΔDC^ mice”). Lung cDCs from XBP1^ΔDC^ mice, ATF6α^ΔDC^ mice and control littermates were analyzed by flow cytometry (Figures 3A–D). We observed that ATF6α^ΔDC^ mice display comparable lung cDC1 percentages with control counterparts (Figures 3A, B). However, these observations were not recapitulated in XBP1^ΔDC^ mice, which revealed an evident loss of cDC1s compared to control littermates, confirming previous findings (Tavernier et al., 2017) (Figures 3C, D). These data indicate that despite reported evidence demonstrating transcriptional regulation of *Xbp1* by ATF6α (Yoshida et al., 2001), the *in vivo* functional outcomes of these transcription factors in tissue DCs do not overlap. To explain these results, we investigated if ATF6α deficient DCs express altered levels of X*bp1* mRNA. PCR analysis of sorted splenic cDC subsets revealed that ATF6α-deficient cDC1s and cDC2s express normal levels of *Xbp1*u and *Xbp1*s (Figure 3E), indicating that ATF6α deficiency does not alter XBP1 expression in tissue cDCs.

We also quantified the composition of additional lung immune cells in ATF6α^ΔDC^ mice. Interestingly, analysis revealed a significant reduction in the frequencies of lung monocytes compared to control mice (Figures 3F, G). Notably, this reduction was not observed in XBP1^ΔDC^ mice (Figures 3H, I), suggesting that ATF6α and XBP1s regulate the fate of myeloid cells by independent mechanisms. These findings prompted us to investigate whether wild-type lung monocytes show signs of ATF6α transcriptional activity in steady state. To this end, we quantified expression of *Atf6* mRNA and the ATF6α targets *Hspa5* (BiP) and *Grp94* by qPCR in isolated lung monocytes from control animals, and transcript levels were compared with those measured in lung T cells (Figure 3M, grey bars). Data indicated that lung monocytes tend to express higher levels of *Atf6*, *Hspa5* and *Grp94* than T cells isolated from the same tissue, suggesting that the former cell type show signs of ATF6α transcriptional activity in the steady state lung.

Finally, to evaluate if the remaining population of lung monocytes from ATF6α^ΔDC^ mice display signs of cellular dysregulation, we measured expression of canonical surface molecules and ATF6α targets by flow cytometry and qPCR, respectively. These cells express normal levels CD11c, MHC-II, CD64 and F4/80 (Figure 3J–L), and were CD11c^-/int^ MHC-II^-/lo/+^ CD64^lo^ F4/80^lo^, in line with the definition of Ly6C^+^ pulmonary monocytes (Gibbings et al., 2017). In addition, lung monocytes from ATF6α^ΔDC^ mice expressed similar levels of *Atf6*, *Hspa5* and *Grp94* than control animals, suggesting that the remanent monocyte population from ATF6α^ΔDC^ mice are not targeted by Cre-mediated recombination (Figure 3M). To sum up, these data suggest that lung monocytes show signs of basal ATF6α transcriptional activity and that ATF6α loss in CD11c^+^ cells result in partial reduction of lung monocyte frequencies.

### ATF6α deficiency in steady state cDCs does not alter expression of UPR components

**FIGURE 4:**
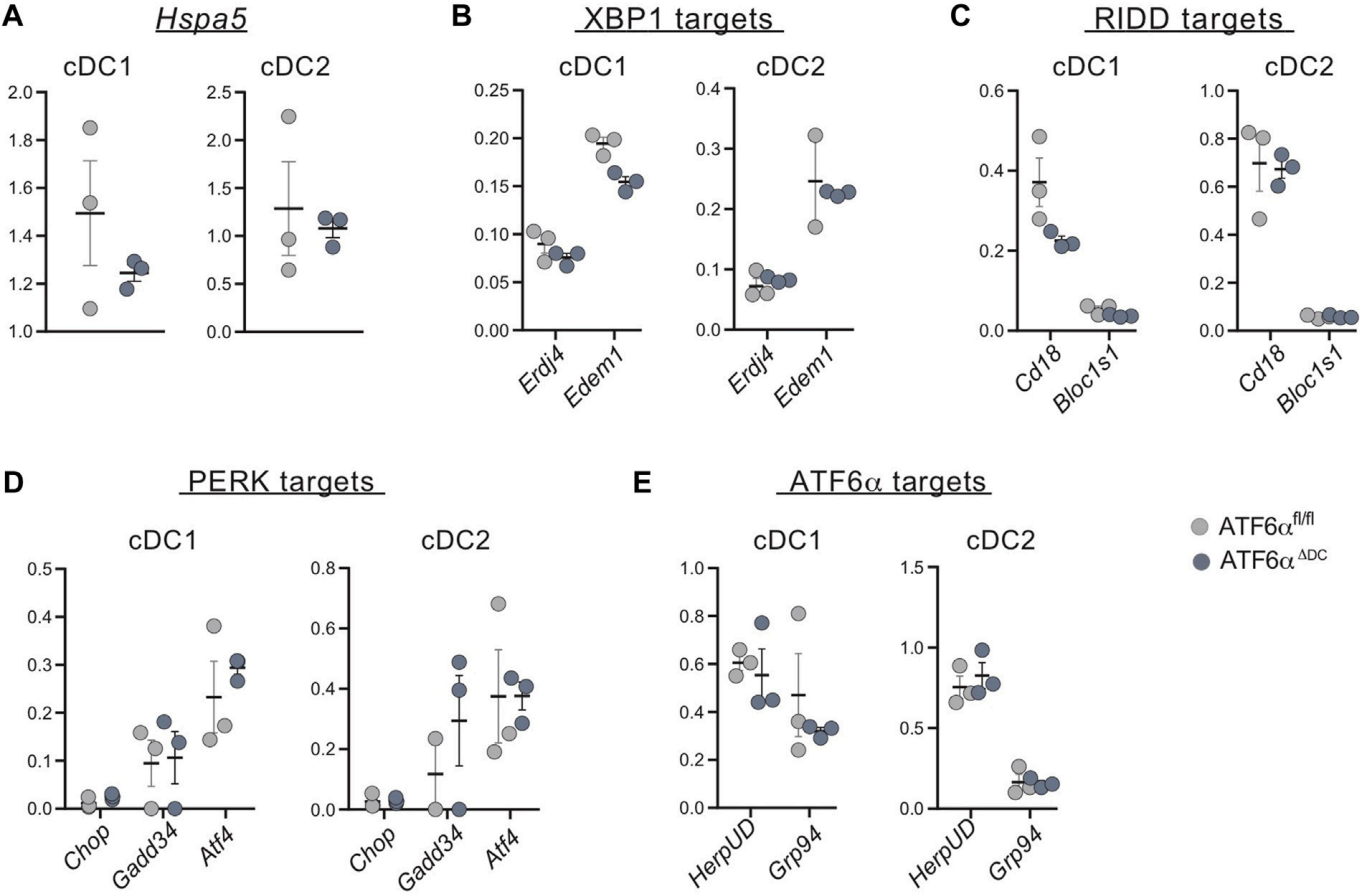
ATF6α deficient cDCs express normal level of UPR components. qPCR analysis of basal expression of UPR target genes from sorted spleen cDCs of ATF6α^fl/fl^ (*n* = 3 mice) and ATF6α^ΔDC^ mice (*n* = 3 mice). **(A)** Expression of *Hspa5* (BiP) was used as a readout of ER stress, and specific target genes of **(B)** IRE1-XBP1 axis, **(C)** RIDD branch, **(D)** PERK and **(E)** ATF6α pathways were quantified by qPCR. RNA expression was normalized to housekeeping genes L27. Each symbol represents the sample obtained from one mouse in two independent experiments. For statistical analyses, the non-parametric Mann-Whitney test was used.

Finally, given that ATF6α does not control expression of *Xbp1*u/s in cDCs (Figure 3E), we sought to investigate whether ATF6α could regulate expression of additional UPR components in these cells. To this end, target genes of the ATF6α, PERK and IRE1 (XBP1s and RIDD targets) branches were quantified by qPCR in cDC1s and cDC2s isolated from spleen of ATF6α^ΔDC^ and ATF6α^WT^ mice (Figure 4). Data show that ATF6α deficient cDC1s express a trend towards reduced *Hspa5* expression, which did not reach statistical significance (Figure 4A). Furthermore, ATF6α deficient cDCs express unaltered levels of the XBP1s targets *Erdj4* and *Edem1* (Figure 4B), the RIDD substrates *Cd18* and *Bloc1s1* (Figure 4C) and the PERK targets *Chop*, *Atf4* and *Gadd34* (Figure 4D). Notably, cDCs from ATF6α^ΔDC^ mice also expressed normal levels of the reported ATF6 targets *HerpUD* and *Grp94* (Figure 4E). These data show that cDCs do not constitutively activate the ATF6α branch in steady state. Furthermore, these observations indicate that in absence of canonical ER stress, ATF6α does not regulate expression of UPR components in cDCs *in vivo*.

### ATF6α regulate the production of the proinflammatory cytokines IL-12 and IL-6 during DC activation

**FIGURE 5:**
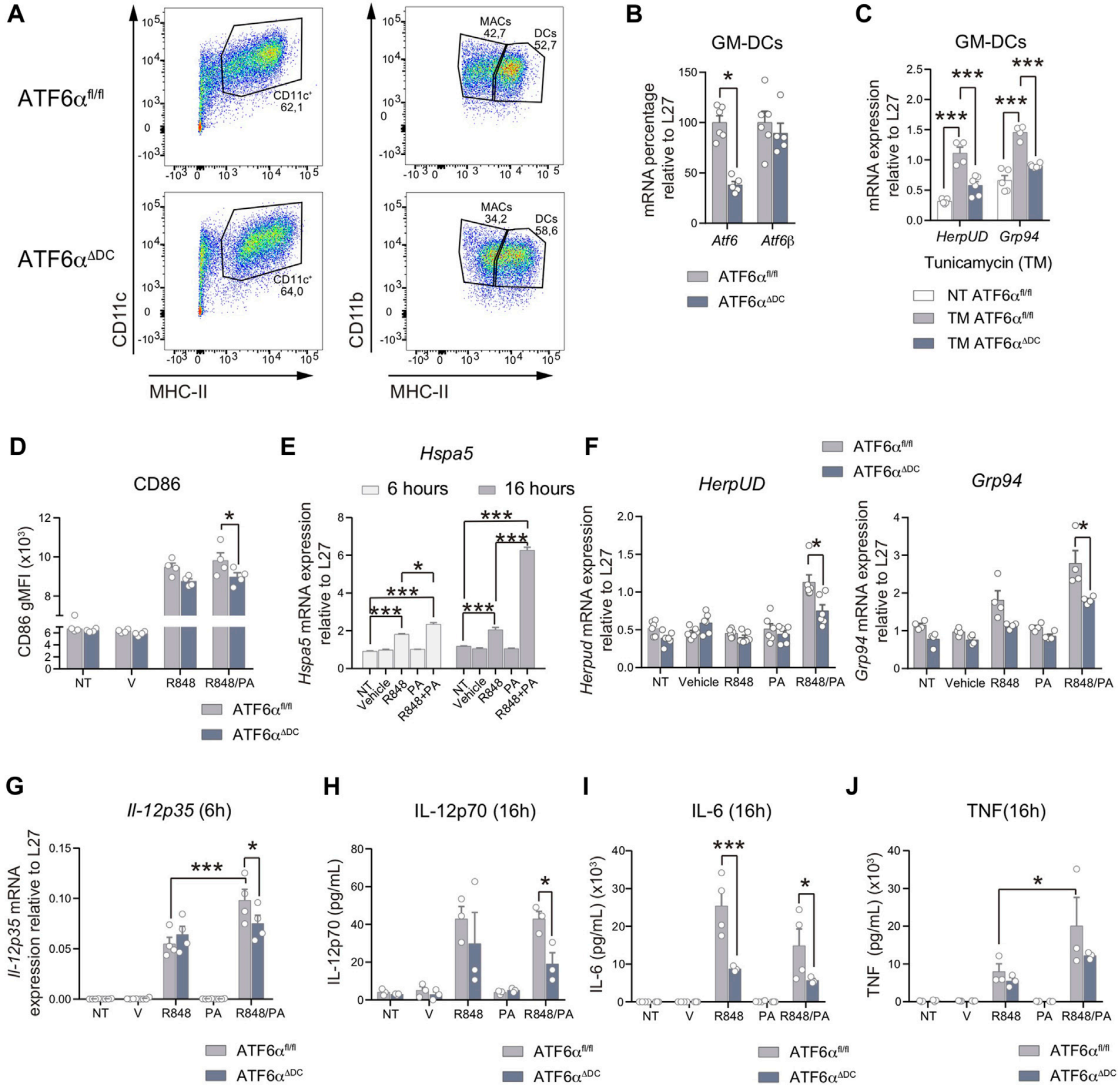
ATF6α regulates IL-12 and IL-6 production by activated DCs **(A)** Representative flow cytometry plots of GM-DCs from ATF6α^fl/fl^ and ATF6α^ΔDC^ mice. Cells were gated as singlets, live, CD11b^+^, CD11c^+^, MHC-II^+^. **(B)** Quantification of *Atf6* and *Atf6b* mRNA in GM-DCs from ATF6α^fl/fl^ and ATF6α^ΔDC^ mice (*n* = 5–6). **(C)** Expression of the ATF6α targets *Herpud* and *Grp94* in ATF6α suficient or deficient GM-DCs stimulated with tunicamycin (TM) (*n* = 3). **(D)** Flow cytometry analysis of CD86 in GM-DCs from ATF6α^fl/fl^ and ATF6α^ΔDC^ mice stimulated with R848 (5 μg/mL) with or without palmitic acid (0,5 mM, PA) for 16 h. GM-DCs were pre-gated as singlets, live, CD11b^+^, CD11c^+^, MHC-II ^high^. **(E)** Expression of *Hspa5* (BiP) mRNA in GM-DCs from ATF6α^fl/fl^ activated with the indicated stimuli for 6 and 16 h. **(F)** Expresion of *Herpud* and *Grp94* in GM-DCs from ATF6α^fl/fl^ and ATF6α^ΔDC^ mice and activated with the indicated stimuli for 24 h (*n* = 4–6). **(G)** *Il-12p35* mRNA expresion in GM-DCs from ATF6α^fl/fl^ and ATF6α^ΔDC^ mice and activated with the indicated stimuli for 6 h (*n* = 4). Measurement of secreted cytokines IL-12p70 **(H)**, IL-6 **(I)**, TNF **(J)** from supernatants of GM-DCs from ATF6α^fl/fl^ and ATF6α^ΔDC^ mice, and stimulated for 16 h (*n* = 3, 4). For statistical analyses, a non-parametric Mann-Whitney test was used, *p < 0.05.

Finally, we sought to evaluate if ATF6α regulates the acquisition of immunogenic features in contexts of DC activation. To this end, we studied bone-marrow derived DCs cultured in presence of the differentiation factor GM-CSF (referred to as ‘GM-DCs’). We corroborate that GM-DC cultures of ATF6α^ΔDC^ mice generate normal proportion of DCs (Figure 5A) and display *Atf6* ablation without interfering with *Atf6b* expression (Figure 5B). To determine ATF6α transcriptional activity, we treated GM-DCs from ATF6α^ΔDC^ and control mice with the pharmacological ER stressor tunicamycin (Figure 5C). As expected, tunicamycin treatment induces activation of the ATF6α targets *HerpUD* and *Grp94*, and expression of these transcripts are reduced in GM-DCs deficient for ATF6α (Figure 5C), validating the model of study. Next, we interrogated if ATF6α contributes to the acquisition of immunogenic features during DC activation. For this purpose, we studied two types of stimuli; R848 (Resiquimod, an imidazoquinoline agonist of toll-like receptor 7 -TLR7 that possesses antiviral activity), and R848 combined with palmitic acid (R848/PA), which is a saturated fatty acid reported to induce activation of XBP1s, ATF4 and CHOP in activated DCs, potentiating their immunogenic function (Mogilenko et al., 2019). Our data indicate that both R848 and R848/PA elicit competent GM-DC activation by means of CD86 expression (Figure 5D). However, only R848/PA induces persistent BiP induction in GM-DCs (Figure 5E), confirming that the mixed stimuli trigger sustained UPR activation in these cells. As reported (Mogilenko et al., 2019), R848/PA also induced efficient activation of XBP1s y PERK branches (Supplementary Figure 4A), so we sought to investigate if R848/PA was also competent to trigger ATF6α transcriptional activity. Data depicted in Figure 5F shows that R848/PA efficiently induce *HerpUD* and *Grp94* expression in GM-DCs in an ATF6α dependent manner, confirming that the mixed stimuli activate the transcription factor in DCs. To connect these findings with a functional role, we investigated if ATF6α regulates cytokine production in activated DCs. GM-DCs from ATF6α^ΔDC^ and control mice were stimulated with R848, R848/PA or vehicle and the production of proinflammatory cytokines was determined on mRNA and protein level. As reported (Mogilenko et al., 2019), PA treatment markedly augments the expression of *Il-23p19* mRNA in R848-stimulated GM-DCs, although we found that expression of the cytokine is not regulated by ATF6α (Supplementary Figure 4B). IL-23 belongs to the IL-12 family of cytokines, in which IL-12 is a broadly studied factor involved in the generation of T helper 1 and natural killer responses, among others (Gee et al., 2009). The bioactive IL-12 form (termed IL-12p70) is comprised of the IL-12p35 and IL-12p40 subunits, in which the latter component is shared with IL-23 (IL-23p19/IL-12p40) (Gee et al., 2009). We observed that R848/PA stimulation also led to a significant increase in *Il-12p35* mRNA expression compared to R848 alone (Figure 5G). Interestingly, compared to control GM-DCs, ATF6α deficient cells show decreased *Il-12p35* mRNA expression upon R848/PA stimulation (Figure 5G). To extend these findings to protein level, we quantified IL-12p70 secreted in the supernatants of activated GM-DCs. Data depicted in Figure 5H shows that ATF6α-deficient GM-DCs stimulated with R848/PA secrete lower levels of IL-12p70 compared to control counterparts. These data indicate that ATF6α regulates IL-12 production by activated DCs. Next, we analyzed expression of additional proinflammatory cytokines and found that IL-6 was also reduced in the supernatants from ATF6α *knock-out* GM-DCs stimulated with R848 or R848/PA (*p*-value = 0.06) (Figure 5I). Notably, we found no regulation of *Il-6* mRNA transcript levels by ATF6α (Supplementary Figure 4C), suggesting regulation of the cytokine on translational/posttranslational level. Furthermore, the regulation of ATF6α on cytokine production was not extended to all proinflammatory cytokines as TNF, another factor produced by activated GM-DCs was not regulated by the transcription factor (Figure 5J; Supplementary Figure 4D). Altogether, these findings demonstrate that ATF6α selectively contributes to the production of IL-12p70 and IL-6 in DCs activated with inflammatory triggers.

## Discussion

ATF6α is a main UPR sensor known for coordinating ER stress responses, which is also emerging as a novel regulator in several pathologies (Hillary and Fitzgerald, 2018). ATF6α roles have been implicated in adipogenesis, neural and muscular embryogenesis, retina development, foveal disease and heart failure, among others (Hillary and Fitzgerald, 2018; Blackwood et al., 2019; Correll et al., 2019; Lee et al., 2020; Kroeger et al., 2021). However, in cells of the immune system, the contribution of ATF6α has not been extensively studied. Here, we studied the role of ATF6α in DCs from tissues and from *in-vitro* cultures, which are known to activate UPR components during development, function, and survival (Osorio et al., 2014; Tavernier et al., 2017; Mendes et al., 2020). Using conditional deficient mice for ATF6α in the CD11c^+^ compartment, we show that loss of the transcription factor does not alter cDC frequencies in lymphoid and non-lymphoid organs, and it does not regulate expression of activation markers in steady state. These findings differentiate ATF6α from the additional UPR sensors IRE1 and PERK, which display specific cellular functions in cDCs (Osorio et al., 2014; Tavernier et al., 2017; Mendes et al., 2020). Furthermore, data obtained with ATF6α conditional deficient mice in DCs did not functionally emulate the loss of XBP1 in DCs, which severely impacts cDC1 survival in the lung (Tavernier et al., 2017). Indeed, ATF6α deficient DCs from spleen express normal levels of *Xbp*1u/s, suggesting that additional mechanisms promote XBP1 expression in DCs. Whether these observations can be explained by functional compensation between ATF6α and ATF6β remains to be confirmed. Even though ATF6α plays protective roles in pathological settings such as those induced by ischemia/reperfusion damage in several organs [(Blackwood et al., 2019), and reviewed in (18)], synergistic effects between ATF6α and ATF6β have also been reported in development (Yamamoto et al., 2007) and cardiac failure settings (Correll et al., 2019). In fact, ATF6β has shown to play overlapping roles with ATF6α in settings of heart hypertrophy (Correll et al., 2019). These data suggest that the interactions and functional outcomes of ATF6α and ATF6β *in vivo* may diverge to the observations made in *vitro* systems. Future work should elucidate whether tissue DC homeostasis is co-regulated by ATF6α/ATF6β interactions. In addition, our data show that ATF6α deficiency did not alter expression of UPR components in DCs, even in targets of ATF6α branch. This evidence indicates that in absence of ER stress, steady state cDCs do not spontaneously activate the ATF6α transcriptional core of genes, differentiating this UPR module from the IRE1/XBP1s and PERK branches (Osorio et al., 2014; Tavernier et al., 2017; Mendes et al., 2020).

An aspect emerging from this work is that ATF6α loss in CD11c^+^ expressing cells resulted in a decrease of monocyte frequencies in the lung. Lung monocytes are a heterogeneous population of cells and a subgroup of pulmonary monocytes are reported to express CD11c (Gibbings et al., 2017). Therefore, the decrease in monocyte frequencies in the lungs of ATF6α^ΔDC^ mice could be due to direct effects in monocyte subtypes targeted by the *Itgax*-Cre mice line, as previous work shows that the mice line display an efficiency of 30% approx. Of Cre-mediated deletion in peripheral blood monocytes (Abram et al., 2014). The question as to why the reduction in monocyte frequencies in ATF6α^ΔDC^ mice is selectively noticed in the lung compared to other tissues remains to be elucidated. Future studies using selective Cre-transgenic lines that allow optimal targeting of the monocyte population will help addressing the contribution of ATF6α in monocyte biology.

Finally, we uncover a novel role for ATF6α in settings of DC activation. Upon stimulation with a mix of TLR ligands and saturated fatty acids, cultured DCs become activated and induce the ATF6α branch of the UPR, which contribute to the production of IL-12. These observations complement previous findings showing that IL-23, another member of the IL-12 family of cytokines, is optimally produced by DCs *via* a XBP1-ATF4-CHOP dependent mechanism (Mogilenko et al., 2019). Our findings identify IL-12 as an additional cytokine produced upon TLR ligand/fatty acid stimulation and identify ATF6α as a regulator of the process. The molecular mechanisms accounting for IL-12 regulation by ATF6α remain to be further investigated, as we did not find canonical ERSE and ERSE-II motifs in the promoter regions of the *Il12a* and *Il12b* genes (data not shown). Furthermore, we also observe that IL-6 production displays ATF6α dependency, even in conditions lacking saturated fatty acids. Importantly, the regulation of cytokine production by ATF6α is not extended to all inflammatory factors, as TNF is not controlled by ATF6α expression. On the other hand, the data presented here suggest that targeting ATF6α may be beneficial in selective contexts of inflammation that evoke an IL-12/IL-6 cytokine response, which may include infection with intracellular bacteria or certain autoimmune contexts (Gee et al., 2009). Understanding the mechanisms underlying selective cytokine production by UPR components is critical to translate these findings to clinically relevant settings. From this work, it emerges the notion that there may be a “division of labor” among UPR sensors in the regulation of cytokine production to inflammatory settings.

## Materials and methods

### Experimental model and subject details

#### Mice

ATF6α^WT^ (ATF6fl/fl (Engin et al., 2013), obtained from The Jackson Laboratory), ATF6α^ΔDC^ (ATF6fl/fl x *Itgax*-Cre (Caton et al., 2007)), XBP1^WT^ (XBP1fl/fl (Lee et al., 2008)), XBP1^ΔDC^ (XBP1fl/fl x *Itgax*-Cre (Caton et al., 2007)), mice were bred at Universidad de Chile and Fundación Ciencia y Vida in specific pathogen-free conditions. All mice were kept on a C57BL/6 background. Litters with mice of both sexes at 10–14 weeks of age were used for experiments.

### Method details

#### Preparation of cell suspensions

Spleens and livers were minced and digested in PBS supplemented with 10% FBS with Collagenase D (1 mg/mL, Roche) and DNAse I (50 μg/mL, Roche) for 30 min at 37°C in a water bath. Digested tissue was then passed through a 70 μm cell strainer, followed by red blood cell lysis with RBC lysis buffer (Biolegend). Single cells were kept on ice.

Lungs were minced and digested in RPMI 1640 with Liberase TM (0.02 mg/mL; Roche) and DNAse I (50 μg/mL, Roche) for 30 min at 37°C in a water bath, resuspending the tissue with help of a Pasteur pipette every 10 min during the incubation. Digested tissue was then passed through a 70 μm cell strainer, followed by red blood cell lysis with RBC lysis buffer (Biolegend). Single cells were kept on ice.

Intestines were cleaned with HBSS and the mesenteric lymph node, fat, and Peyer’s patches were removed. Tissue was incubated for 20 min at 37°C with constant stirring at 100 rpm in RPMI 1640 medium supplemented with 1M DTT (Thermo Fisher Scientific), 0,5 M EDTA (Thermo Fisher Scientific). Tissues were minced and incubated in RPMI 1640 medium supplemented with liberase TL 12,5 mg/mL, (Roche), DNAse I 10 mg/mL for 30 min at 37°C with constant stirring at 100 rpm and then smashed through a 40 mm sterile strainer. SiLP cells were centrifuged (700 g, 20 min, 25°C) in 2 step percoll (GE Healthcare) gradients (40% and 75%). Leukocytes were enriched in the 40%–75% edge fraction.

For cDCs sorting, spleens were minced and digested as previously described and the single-cell suspension was enriched prior to cell sorting by depletion of CD3e and B220 expressing cells using biotin-labeled monoclonal antibodies, anti-biotin microbeads and isolation kits (Miltenyi Biotec).

#### Flow cytometry and cell sorting

For surface staining, cells were incubated with anti-Fc receptor antibody and then stained with fluorochrome-conjugated antibodies in FACS buffer (PBS + 1% FBS + 2 mM EDTA) for 20 min at 4°C. Viability was assessed by staining with fixable viability Zombie UV (BioLegend). A biotinylated antibody was used for F4/80 staining, followed by a second staining step with Streptoavidin-BUV737 (BD Biosciences) for 20 min at 4°C. Flow cytometry was performed on BD LSR Fortessa (BD Biosciences) instruments using FACSDiva software (BD Biosciences). Analysis of flow cytometry data was done using FlowJo software. Cell sorting was performed using FACS Aria III (BD Biosciences). Antibody clones used in this study are illustrated in Supplementary Methods.

#### RNA isolation, cDNA generation and qPCR analysis

Total RNA was extracted from sorted spleen cDCs, lung monocytes and T cells with the RNeasy Micro Kit (Qiagen) following manufacturer’s instructions. Total RNA from GM-DCs was extracted using TRizol reagent (Invitrogen). cDNA was prepared using M-MLV reverse transcriptase (Invitrogen). qPCR was performed with a SYBR Green PCR Master Mix kit (Applied Biosystems). (See Supplementary Methods for primers used for qPCR).

#### Xbp1s splicing assay

Total RNA was isolated by RNeasy plus Micro Kit (Qiagen) following manufacturer’s instructions. cDNA was prepared using M-MLV reverse transcriptase (Invitrogen). The following primers were used for conventional PCR amplification of total *Xbp1* spliced and *Xbp*1 unspliced: Fwd: 5′-ACA​CGC​TTG​GGA​ATG​GAC​AC-3′ and Rev: 5′-CCA​TGG​GAA​GAT​GTT​CTG​GG-3’ (Martinon et al., 2010); and for beta actin (*Actb*): Fwd: 5′-CTA​AGG​CCA​ACC​GTG​AAA​AG-3′ and Rev: 5′-TTG​CTG​ATC​CAC​ATC​TGC​TG-3’. PCR products were analyzed on 2.8% agarose gels.

#### Bone marrow-derived DC cultures (GM-DCs)

3 × 10^6^ bone marrow cells were seeded in 10 mL of complete medium (RPMI 1640 glutaMAX (Gibco), supplemented with penicillin/streptomycin (100 μg/mL, Corning), 2-mercaptoethanol (50 μM, Gibco), 10% heat-inactivated fetal bovine serum (Hyclone), and recombinant GM-CSF (20 ng/mL, Biolegend). Cells were incubated at 37°C in 5% CO_2_. 0n day 3, 10 mL of complete medium containing GM-CSF (20 ng/mL) was added to the plate. On day 6, half of the medium was removed, and it was replaced by fresh medium supplemented with GM-CSF. Cells (GM-DCs) were harvested on day 9 and used for experiments.

#### Activation of GM-DCs

GM-DCs were activated with R848 (5 μg/mL, Invivogen) with or without palmitic acid (PA, 0.5 mM, Sigma) conjugated with BSA (molar ratio PA:BSA 6:1, Sigma). Controls were RPMI (non-treated, NT), vehicle (BSA 0.083 mM with 0,5 mM ethanol). For cytometric bead array (CBA) assay (BD Biosciences), GM-DCs were cultured at 1 × 10^6^/mL in complete medium for 16 h. The supernatant was collected, and cytokines were quantified following manufacturer’ instructions. For UPR activation with tunicamycin, GM-DCs were cultured as above, and cells were stimulated with tunicamycin (1 μg/mL, Sigma) for 8 h.

### Quantification and statistical analysis

Statistical analysis was conducted using GraphPad Prism software (v9.1.2). Results are presented as mean ± SEM. Two groups were compared using non-parametric two-tailed Mann-Whitney test as indicated in figure legends. A *p*-value < 0.05 was considered statistically significant.

### Study approval

All animal procedures were approved and performed in accordance with institutional guidelines for animal care of the Fundación Ciencia y Vida and the Faculty of Medicine, University of Chile, and were approved by the local ethics committee.

## Data Availability

The raw data supporting the conclusion of this article will be made available by the authors, without undue reservation.
