# Supplementary material for: Normal tissue homeostasis and impairment of selective inflammatory responses in dendritic cells deficient for ATF6α
Source: Front Cell Dev Biol. 2023 Mar 21;11:1089728. doi: 10.3389/fcell.2023.1089728 (PMC10070697; doi:10.3389/fcell.2023.1089728)
Supplement: Supplementary file 1 [file DataSheet1.pdf]

Supplementary Methods

Table I. Probes and antibodies

Antibody	Fluorophore	Clone	Dilution	Brand
**Markers for identification of immune populations**				
XCR1	PE	ZET	1/400	Biolegend
B220	PE-Cy5	RA3-6B2	1/1600	Biolegend
CD3	PE-Cy5	145-2C11	1/400	Biolegend
Sirp-α	PE-Cy7	P84	1/400	Biolegend
CD103	PE-Cy7	2E7	1/200	Biolegend
CD11c	APC	N418	1/800	eBioscience
CD45	AlexaFluor700	30-F11	1/1600	Biolegend
Ly6G	AlexaFluor700	1A8	1/800	Biolegend
MHC-II	APC-Cy7	M5/114.15.2	1/1600	Biolegend
CD11b	Brilliant Violet 421	1/800	M1/70	Biolegend
B220	Brilliant Violet 510	1/400	RA3-6B2	Biolegend
CD3	Brilliant Violet 510	1/200	145-2C11	Biolegend
Ly6C	Brilliant Violet 605	HK1.4	1/200	Biolegend
NK1.1	Brilliant Violet 650	PK136	1/100	Biolegend
XCR1	Brilliant Violet 650	ZET	1/400	Biolegend
CD64	Brilliant Violet 711	X54-5/7.1	1/100	Biolegend
CD11c	Brilliant Violet 711	N418	1/800	Biolegend
CD11b	Brilliant Violet 785	M1/70	1/800	Biolegend
CD45	Brilliant Ultraviolet 395	30-F11	1/800	BD Biosciences
Streptavidin	Brilliant Ultraviolet 737	-	1/100	BD Biosciences
F4/80-Biotina	-	BM8	1/100	Biolegend
B220-Biotina	-	RA3-6B2	1/100	Biolegend
CD3-Biotina	-	145-2C11	1/200	Biolegend
**Markers for costimulatory molecule identification**				
CD274 (PD-L1)	PE	MIH5	1/200	Invitrogen
CD86	PE-Cy7	GL-1	1/400	Biolegend
CD40	APC	3/23	1/200	Biolegend
**Viability dye and Fc blocking**				
Zombie UV	BUV496	-	1/500	Biolegend
Fc- Block (CD16/32)	-	-	1/400	Biolegend

Table II. qPCR primers

Gene		Primer sequence	Ref
**L27**	Fwd	GCCAAGCGATCCAAGATCAA	Probe 128 UPL*
	Rev	GCTGGGTCCCTGAACACATC	
**Atf6α**	Fwd	ACCATGTGGTGAATGTGCTG	(1)
	Rev	TTCTTCTTGCGCGACTGACA	
**Atf6β**	Fwd	ACTAACCACAGTTCCGGTGC	(1)
	Rev	GGCATAGGGGCTGGAACAAT	
**Bip (Hspa5)**	Fwd	ATGAGGCTGTAGCCTATGGTG	Probe 64 UPL*
	Rev	GGGGACAAACATCAAGCAG	
**Erdj4**	Fwd	CCCCAGTGTCAAACTGTACCAG	(2)
	Rev	AGCGTTTCCAATTTTCCATAAATT	
**Edem1**	Fwd	AAGCCCTCTGGAACTTGCG	(3)
	Rev	AACCCAATGGCCTGTCTGG	
**Cd18**	Fwd	AACGAGATCACCGAGTCTGG	(4)
	Rev	CAGGCCTTCTCCTTGTTGG	
**Bloc1s1**	Fwd	CACCCAGCCAGACTCGAC	(4)
	Rev	GCAGCGATAGCTTCTCTCCTC	
**Chop**	Fwd	CCACCACACCTGAAAGCAG	Probe 33 UPL*
	Rev	TCCTGCAGATCCTCATACCAG	
**Gadd34**	Fwd	GAGATTCCTCTAAAAGCTCGG	(5)
	Rev	CAGGGACCTCGACGGGCAGC	
**Atf4**	Fwd	ATGGCCGGCTATGGATGAT	(5)
	Rev	CGAAGTCAAACTCTTTCAGATCCATT	
**Herpud**	Fwd	AGCAGCCGGACAACTCTAAT	(5)
	Rev	CTTGGAAAGTCTGCTGGACA	
**Grp94**	Fwd	AATAGAAAGAATGCTTCGCC	(5)
	Rev	TCTTCAGGCTCTTCTTCTGG	

UPL: Universal ProbeLibrary

REFERENCES

1. Sharma RB, Darko C, Alonso LC. Intersection of the ATF6 and XBP1 ER stress pathways in mouse islet cells. J Biol Chem [Internet]. 2020;295(41):14164–77.

2. Lee A, Scapa E, Cohen D, Glimcher L. Regulation of hepatic lipogenesis by the transcription factor XBP1. Science. 2008;320(5882):1492–6.

3. Hetz C, Bernasconi P, Fisher J, Lee AH, Bassik MC, Antonsson B, et al. Proapoptotic BAX and BAK modulate the unfolded protein response by a direct interaction with IRE1α. Science (80-). 2006;312(5773):572–6.

4. Osorio F, Tavernier SJ, Hoffmann E, Saeys Y, Martens L, Vetters J, et al. The unfolded-protein-response sensor IRE-1α regulates the function of CD8α + dendritic cells. Nat Immunol. 2014;15(3):248–57.

5. Wu J, Rutkowski DT, Dubois M, Swathirajan J, Saunders T, Wang J, et al. ATF6α Optimizes Long-Term Endoplasmic Reticulum Function to Protect Cells from Chronic Stress. Dev Cell. 2007;13(3):351–64.
