# Supplementary material for: Normal tissue homeostasis and impairment of selective inflammatory responses in dendritic cells deficient for ATF6α
Source: Front Cell Dev Biol. 2023 Mar 21;11:1089728. doi: 10.3389/fcell.2023.1089728 (PMC10070697; doi:10.3389/fcell.2023.1089728)
Supplement: Supplementary file 2 [file Image1.pdf]

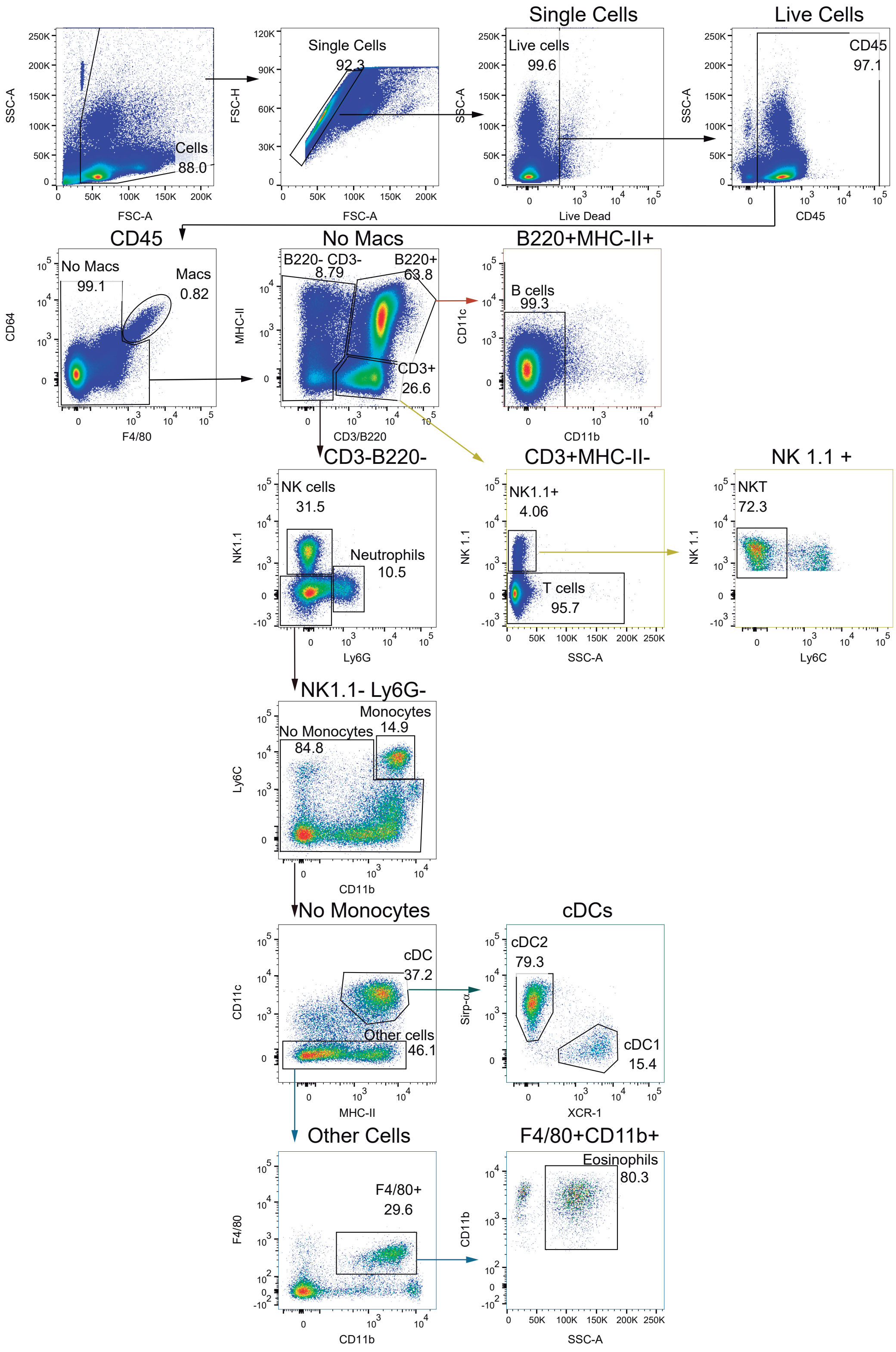

Supplementary Figure 1. Gating strategy for spleen resident immune cells. Representative flow cytometry plots showing identification of myeloid cells such as cDCs, macrophages, monocytes, neutrophils and eosinophils and lymphoid cells such as B cells, T cells, NK and NKT cells residents from the spleen tissue of ATF6$\alpha^{fl/fl}$ mice.

A

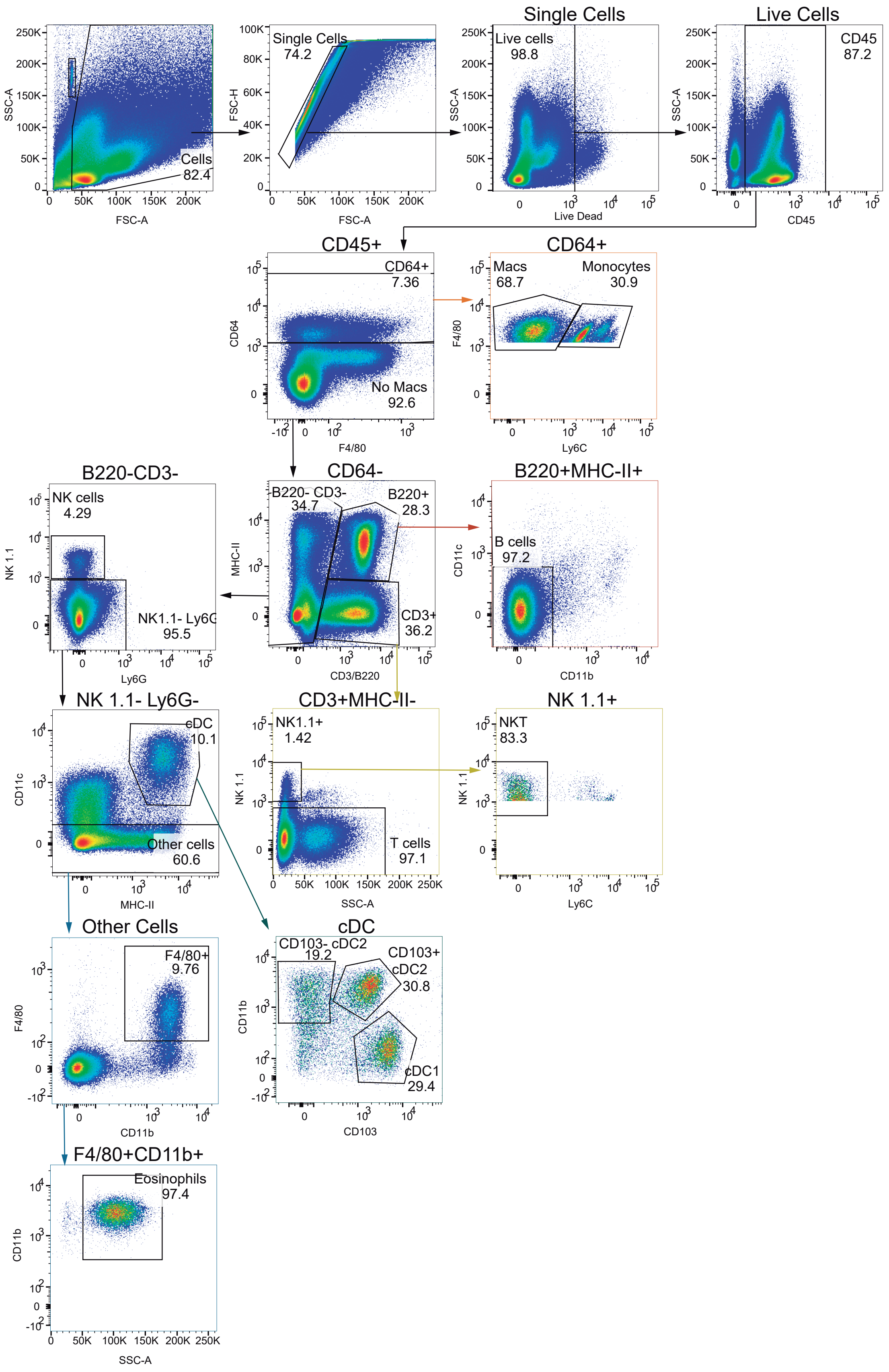
Cells
82.4
FSC-A
SSC-A
Single Cells
74.2
FSC-H
Single Cells
Live cells
98.8
Live Dead
Live Cells
CD45
87.2
CD45+
CD64+
7.36
No Macs
92.6
CD64
F4/80
CD64+
Macs
68.7
Monocytes
30.9
Ly6C
B220-CD3-
NK cells
4.29
NK1.1- Ly6G
95.5
NK 1.1
Ly6G
CD64-
B220- CD3-
34.7
B220+
28.3
CD3+
36.2
MHC-II
CD3/B220
B220+MHC-II+
B cells
97.2
CD11c
CD11b
NK 1.1- Ly6G-
cDC
10.1
Other cells
60.6
CD3+MHC-II-
NK1.1+
1.42
T cells
97.1
SSC-A
NK 1.1+
NKT
83.3
Other Cells
F4/80+
9.76
cDC
CD103- cDC2
19.2
CD103+
cDC2
30.8
cDC1
29.4
CD103
F4/80+CD11b+
Eosinophils
97.4

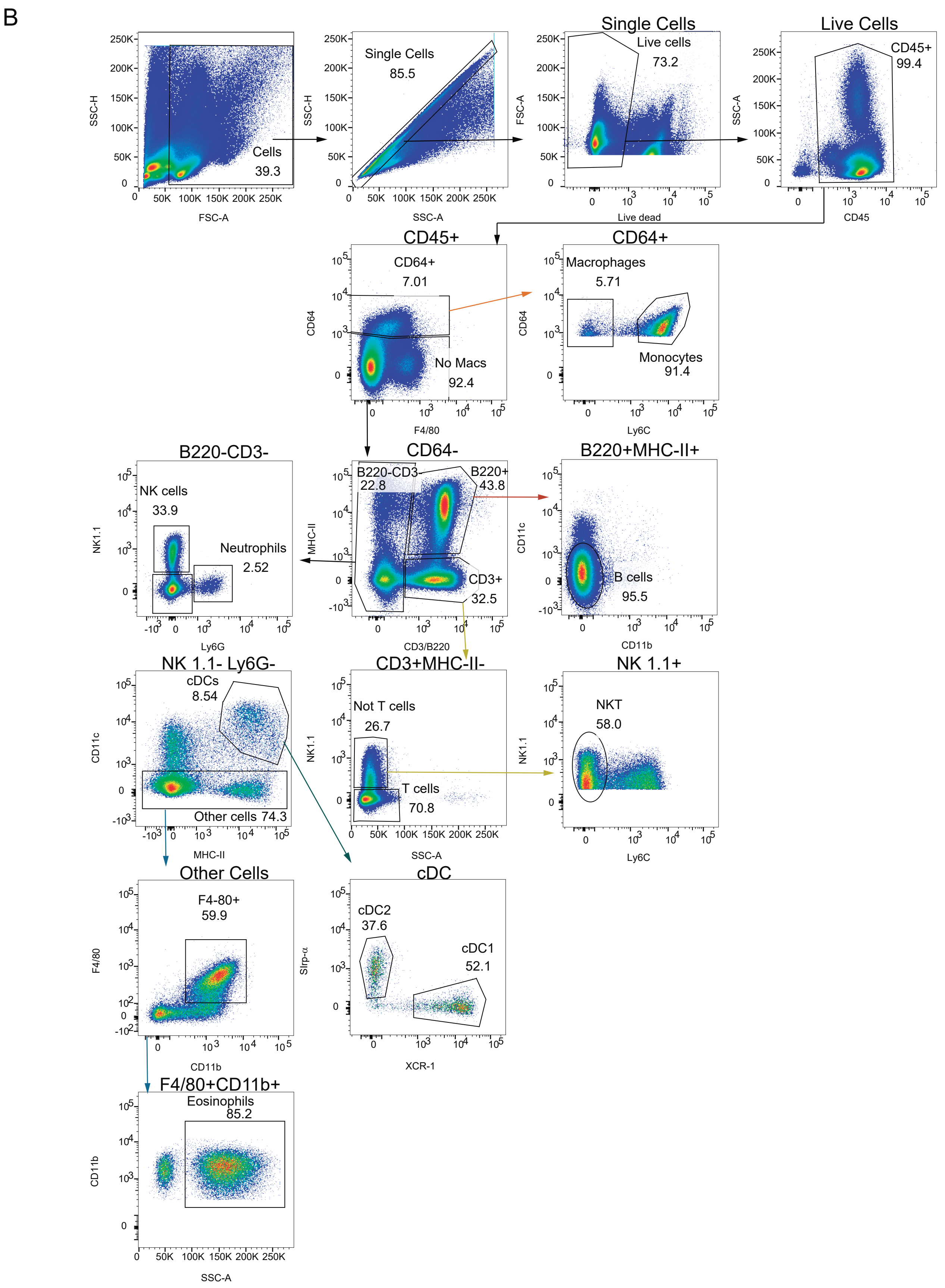

Supplementary Figure 2. Gating strategy for SiLP and liver resident immune cells. Representative flow cytometry plots showing identification of myeloid cells such as cDCs, macrophages, monocytes, neutrophils and eosinophils and lymphoid cells such as B cells, T cells, NK and NKT cells residents from **(A)** SiLP and **(B)** liver tissue of ATF6$\alpha^{fl/fl}$ mice. We used a unique gating strategy for all tissues, only one different marker (CD103 instead XCR1) was used in the identification of cDCs in the small intestine lamina propria.

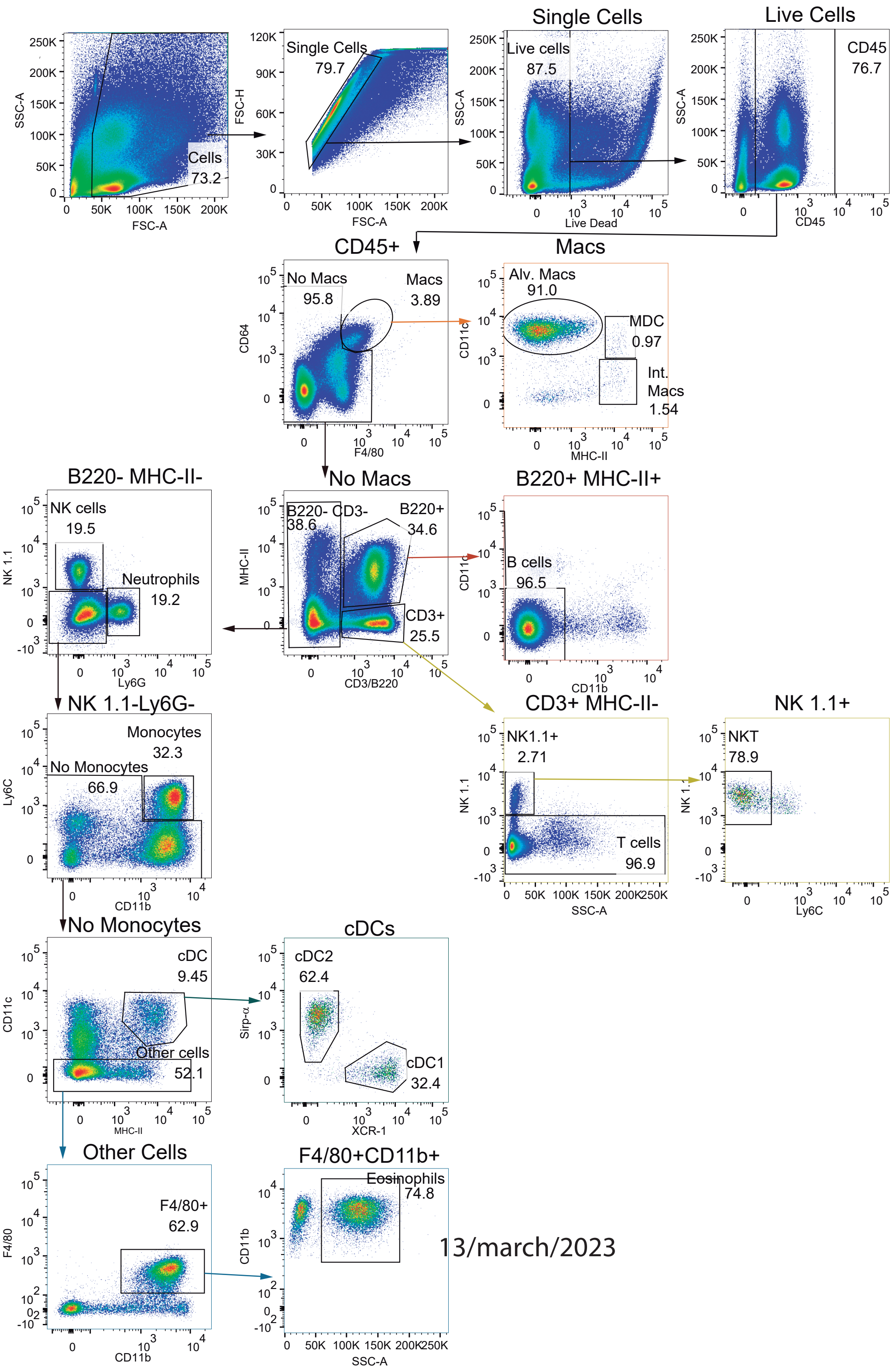

Supplementary Figure 3. Gating strategy for lung resident immune cells.

Representative flow cytometry plots showing identification of myeloid cells such as cDCs, macrophages, monocytes, neutrophils and eosinophils and lymphoid cells such as B cells, T cells, NK and NKT cells residents from the lung tissue of ATF6$\alpha^{fl/fl}$ mice. Macrophages was also distinguished as Alv. Macs: Alveolar macrophages, MDC: Monocytes-derived cells and Int. Macs: Intestitial macrophages.

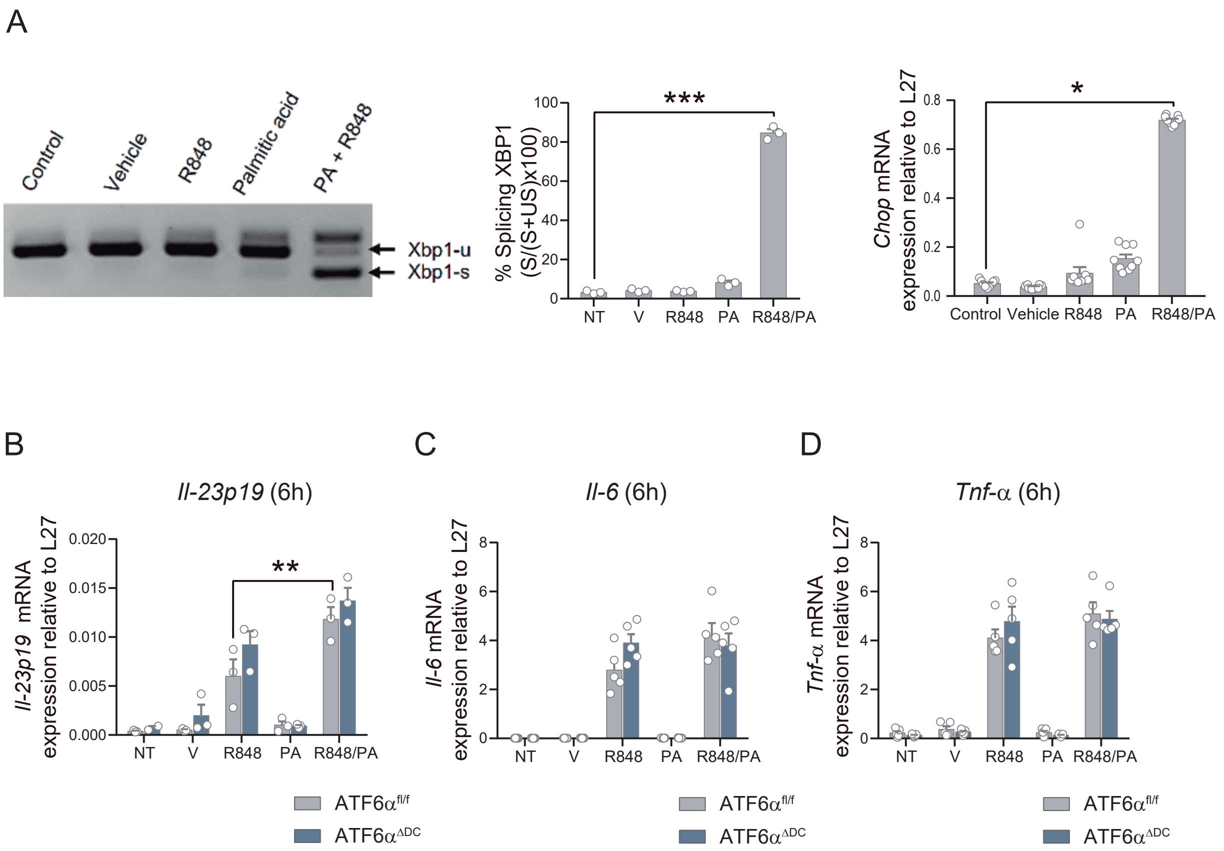

Supplementary Figure 4: Activation of UPR members and functional components in bone-marrow derived DCs stimulated with R848 and PA.

(A) Expression of Xbp1s and Chop by R848/PA-stimulated GM-DCs from control animals. Representative electrophoresis of Xbp1 unspliced and spliced PCR products (left panel, n=3) and quantification of Xbp1s expression (middle panel, n=3) in DCs activated with the indicated stimuli. Chop mRNA expression in GM-DCs activated with the indicated stimuli (right panel, n=3). Expression of Il-23p19 **(B)**, Il-6 **(C)** and Tnf **(D)** in GM-DCs from ATF6αfl/fl and ATF6αΔDC mice and activated with the indicated stimuli for 6h. Data in figures is from three (B) or five (C-D) independent experiments. For statistical analyses, a non-parametric Mann-Whitney test was used, *P < 0.05.
